# Vaginal temperature of lactating cows during heat waves or normal summer day and effect of additional daily cooling treatments as heat load mitigation strategy

**DOI:** 10.1007/s00484-023-02600-6

**Published:** 2023-12-26

**Authors:** A. Vitali, G. Grossi, N. Lacetera

**Affiliations:** Dipartimento Scienze Agrarie e Forestali, Università della Tuscia, Viterbo, Italy

**Keywords:** Dairy cows, Heat waves, Vaginal temperature, Heat load, Cooling treatments

## Abstract

The vaginal temperature (*VT*) of lactating Holstein cows was monitored in not heat wave (*NHW*) and in heat wave (*HW*) summer days. Temperature humidity index (*THI*) was monitored and assigned to four classes of heat load (*HL*): *THI* < 68 null; 68 < *THI* < 74 low; 74 < *THI* < 80 moderate; and *THI* > 80 high.

Five daily treatments consisting of continuous forced ventilation and sprayed water (1′ on follow by 5′ off) were assumed as control cooling protocol (CC) and compared with two experimental cooling protocols (EC) applied in the feed bunk and based on the CC plus two additional cooling treatments which lasted a total of 90′ (EC90) or 150′ (EC150) in the day.

Sixty lactating cows were enrolled in two summer trials carried out in *NHW* or *HW*. In each trial, 10 cows were cooled by CC, 10 by EC90 and 10 by EC150. Twenty additional cows were monitored in a fall trail to have reference value of *THI* and *VT* under thermoneutral conditions (*TN*). Each trial lasted 72 h, and measurements of *VT* were carried out by intra-vaginal data loggers.

The 33% of observed *THI* was within the high class of *HL* during *HW*, whereas *THI* never exceeded the upper threshold of moderate or low class of *HL* in *NHW* and *TN*, respectively.

Multiparous and high yielding cows were more sensitive to *HL*, and the increased daily cooling treatments reduced heat load during hot conditions. However, during heat waves a certain degree of hyperthermia occurred even with intensive cooling management based on seven daily treatments.

## Introduction

Hyperthermia occurs when the animal cannot dissipate the heat accumulated as the result of heat produced for maintenance, production, reproduction, physical activity, etc. and the heat gained from the environment. This condition causes an increase in body temperature and may prompt physiological and behavioural responses (Bernabucci et al. 2014). Physiological responses are aimed at dissipating heat from the body through the increase in respiration rate, peripheral vasodilatation and sweating, whereas behavioural strategies, such as increasing drinking water or reducing feed intake and activity, are aimed at reducing heat gain (Polsky and von Keyserlingk 2017).

Long lasting conditions of heat load (*HL*) negatively affect production (Nardone et al. 2010), reproduction (Biffani et al. 2016) and health (Vitali et al. 2015, 2020) of dairy cows. Furthermore, West (2003) reported that parity and milk yield can affect the susceptibility of dairy cows to heat, in that multiparous and high yielding cows would be more susceptible to heat when compared to primiparous or low yielding subjects, respectively.

The number of days with temperature humidity index (*THI*) above thermoneutral (*TN*) condition (> 68) is increasing in European countries located within the temperate zone (Silanikove and Koluman 2015). Climatologists forecasted an increase in the frequency, intensity and length of heat waves, an extended period of hot weather relative to the expected conditions of the area at that time of year (Beniston et al. 2007). Compared to 2000, by the end of century, risk of severe heat stress is projected to increase for all livestock species in many parts of the tropics and some of the temperate zones and to become climatically more widespread (Segnalini et al. 2013; Thornton et al. 2021).

Different strategies aimed at maintaining normothermia in dairy cattle have been developed and applied at farm level, and they are based on two main approaches: indirect and direct cooling (Roth 2020). Indirect approaches are aimed at reducing heat load on the animals and include shades, increasing natural ventilation, reducing ambient temperature by water and evaporative pads. Instead, the direct ones boost the physiological mechanism of evaporative heat loss that is represented mainly by the evaporation of water from the skin surface. Wetting the coat of the animal and sub-sequent evaporation of water from it by forced ventilation is an evaporative heat loss system introduced by Flamenbaum and colleagues that resulted highly efficient in reducing HL in milking cows (Flamenbaum et al. 1986).

In the last decades, several studies focused on strategies to improve the effectiveness of evaporative cooling operations. Increasing the daily frequency of cooling treatments, spraying cows for longer time or reducing the time of sprinklers off may have a positive effect in the reduction of body temperature and respiration rate in dairy cows (Avendaño-Reyes et al. 2010; Tresoldi et al. 2018; Pinto et al. 2019).

However, to the best of our knowledge, no studies have compared the impact of different degrees of hot conditions, moderate in normal summer day and severe during heat waves, on body temperature of lactating cows considering their parity and production level. It has not either been verified whether the increase of daily cooling treatments may be a valid strategy to mitigate the HL during heat waves.

Therefore, the present study was aimed at investigating the impact of hot conditions in not heat waves or heat waves summer days (*HW* or *NHW*) on vaginal temperature (*VT*) of lactating dairy cows and to evaluate whether the augmentation of daily cooling treatments may be a valid mitigation strategy to reduce heat load.

## Material and methods

### Animals and housing

The study was carried out from June to October 2019 in a commercial farm located 30 km north-west of Rome, and with 1050 to 1200 lactating Holstein cows.

Cows were housed in a free-stall with high roof and all sides open, milked three times per day (at 05:00–09:00 AM, 01:00–05:00 PM and 09:00 PM–01:00 AM) and fed TMR after milking operations. Both the holding area of the milking parlour and the feed bunk were equipped with cooling facilities represented by circular fans (AG 900 RR Ventilators, 0.9 m of diameter and airflow rate 22.250 m^3^/h, Arienti, Italy) and array of sprinklers for micro-irrigation. Fans were 3-m high, 5 m apart, and with the potential of producing forced ventilation of approximately 3 m/s of air at cow level. The array of sprinklers was 1 m above the cows’ back, spaced 1.5 m apart and capable of spraying water at a rate of 12 L/min.

A cohort of eighty cows was enrolled in the study. The animals were monitored in three distinct trials, two carried out in summer (*n* = 60) during heat load (*HL*) conditions and one carried out in fall (*n* = 20) during thermo-neutral (*TN*) conditions.

### Climate condition

The impact of hot conditions on vaginal temperature (*VT*) of cows was evaluated through two distinct summer trials carried out during *NHW* or *HW*. Both *NHW* and *HW* were identified based on forecasted alert bulletins for heat stress released by the Italian Ministry of Public Health for the geographic area of the farm. The system provided four risk levels of heat stress for public health in relation to weather conditions: green colour indicated no risk; yellow colour indicated low risk; orange colour indicated moderate risk; and red colour indicated heat waves with severe risk. Although the service works to guarantee public health, we decided to adopt it as a proactive alert system for predicting different degrees of hot conditions in dairy cows and verified the effect of alternative cooling protocols on *HL*. In practice, bulletins were checked daily and the *NHW* or *HW* trials were organised and performed based on forecasted orange or red alert of heat stress, respectively.

However, to establish the climate condition to which animals were actually exposed during the trials, air temperature and relative humidity were recorded every 5 min by data loggers 174H (TESTO, DE) installed 3-m high in different points of the barns. Air temperature and relative humidity data were thus utilized for calculation of the temperature humidity index (*THI*) according to the following formula of Kelly and Bond, as reported by Ingraham et al. (1979):

$$THI=\left(1.8\times AT+32\right)-\left(0.55-0.55\times RH\right)\times \left[\left(1.8\times AT+32\right)-58\right]$$

where *AT* was the air temperature expressed as °C, and *RH* the relative humidity expressed as fraction of the unit.

To categorize the degree of *HL* during the trials, the values of *THI* were thus assigned to four classes of *HL* based on specific thresholds. The thresholds and classes adopted in the study were: *THI* < 68 as null *HL*; 68 < *THI* < 74 as mild *HL*; 74 < *THI* < 80 as moderate *HL*; *THI* > 80 as high *HL*. The *THI* of 68 was indicated as the threshold above which respiration rate starts to increase (Pinto et al. 2019), and here was considered as the thresholds above which *HL* started. *THI* of 74 and 80 were instead the thresholds provided for Italian dairy cows above which milk yield decreased and mortality risk increased, respectively (Vitali et al 2009; Bernabucci et al. 2014).

### Cooling operations

Cooling operated in the farm was based on continuous forced ventilation with sprinklers 1 min on and followed by 5 min off for a cycle of 6 min (spraying cows 10 min/h). The cooling protocol applied by the farm consisted of 5 daily treatments lasting approximately 1 h each. Three of these treatments were applied in the holding area of the milking parlour where cows stationed approximately 60′ before milking. After milking, cows were moved to their own pen to go directly to the feed bunk where the cooling system was turned on after the first cows arrived. We estimated 60′ as the time spent at feed bunk to eat, and therefore the assumption was that cows were cooled for approximately 1 h. Sprinklers and fans were applied together in the feed bunk only after morning and afternoon milking, whereas after night milking cows were only wet by sprinklers. This cooling protocol represented the control cooling protocol (CC) of the study.

### Experimental design

The control cooling protocol (CC) described above was compared with two experimental cooling protocols (EC) both during summer trials in *NHW* and in *HW*. The two EC were based on the CC plus two additional cooling treatments (continuous forced ventilation with 1 min of wetting followed by 5 min off) applied at 10:30 AM and at 6:30 PM in the feed bunk. The additional cooling treatments lasted in total 90′ (EC90) or 150′ (EC150) as the sum of two distinct treatments lasting 45′ or 75′ each, respectively.

A total of 60 cows belonging to three distinct pens of the farm were enrolled in the two summer trials carried out in *NHW* (no. of cows = 30) or in *HW* (no. of cows = 30). The three pens were equal in terms of dimensions, number of cows and cooling equipment and randomly assigned to CC, EC90 or EC150. During each trial (*NHW* and *HW*) the ten selected cows belonging to EC90 or EC150 pen were walked to the feed bunk at 10:30 AM and at 6:30 PM, locked and cooled for 45′ or 75′, respectively. At these times, the selected cows belonging to CC pen were left free to carry out their voluntary activities (ruminating, resting, eating, etc.) without any extra cooling. The six subgroups of cows monitored, three in *NHW* and three in *HW*, were homogeneous (*mean* ± *SD*) for milk yield (38.0 ± 4.8 L/day), *DIM* (151 ± 75 days) and number of lactations (1.8 ± 1.0).

To evaluate the impact of *HL* in relation to parity and production level, cows were classified for parity as primiparous and multiparous (second parity and above) and categorised as low or high yielding cows corresponding to daily milk yield below or above average daily milk yield of farm (38 L/cow/day), respectively. With regard to parity and milk yield, the six subgroups were arranged with five primiparous and five multiparous cows, and with five low and five high yielding cows, respectively.

Finally, a third trial was carried out during fall days (October) to obtain reference values of vaginal temperature (*VT*) under thermo-neutral (*TN*) conditions. In this trial, 20 cows homogeneous with the summer subgroups for milk yield, parity and *DIM* were monitored with no cooling. Also this group was balanced for number of primiparous (*n* = 10) and multiparous (*n* = 10) and for number of low (*n* = 10) and high (*n* = 10) yielding cows. Climate conditions were verified by measurements of the *THI* as reported above. Both summer and fall trials lasted 72 h.

Values of milk yield, *DIM* and parity of the groups of cows that were compared during the trial are reported in Table 1.

**Table 1:** Mean values (± *SD*) of milk yield, days in milk (*DIM*) and parity of groups of cows engaged during heat wave days (*HW*), not heat wave days (*NHW*) and thermoneutral days (*TN*) trials

Climate	Cooling treatment	Milk yield	*DIM*	Parity
*HW*	CC	38.9 ± 5.3	154 ± 51	2.0 ± 1.2
	EC90	39.1 ± 5.7	139 ± 90	1.9 ± 1.2
	EC150	38.4 ± 4.1	149 ± 77	1.7 ± 0.9
*NHW*	CC	38.3 ± 5.1	153 ± 55	1.8 ± 0.9
	EC90	37.8 ± 5.0	139 ± 77	1.7 ± 0.8
	EC150	38.3 ± 4.8	141 ± 77	1.5 ± 0.6
*TN*	NA	37.4 ± 4.2	163 ± 82	2.0 ± 1.3

Control cooling (CC) protocol based on five daily treatments; experimental cooling protocol EC90 based on CC plus two extra daily cooling treatments lasting in total 90′; experimental cooling protocol EC150 based on CC plus two extra daily cooling treatments lasting in total 150′

*NA* not applicable

### Vaginal temperature measures

The *VT* was recorded in continuous every 5 min for 72 h along trials performed in *HW*, *NHW* and *TN* by using mini temperature data loggers DS1922L (iButtonLink, US) with a temperature range of − 40 °C to + 85 °C. Data loggers were thus connected to computer with Viewer Package (iButtonLink, USA), a combination of iButton software and hardware used to view, configure and collect data. Data loggers were programmed (same start time) and thereafter inserted into the cow’s vagina by using exhausted Controlled Internal Drug Release (CIDR) device. In practice, activated data loggers were inserted into rubber balloons that were tied to the CIDRs, which in turn were inserted into the cows’ vagina by special applicator. At the end of each trial, CIDRs were removed and mini data loggers were recovered, connected to computer, and data of *VT* were downloaded and organised in a database for analysis. The value of 39 °C of *VT* was assumed as the upper threshold of normothermia for lactating Holstein cows involved in this study (Kendall and Webster 2009).

### Statistical analysis

Variation of *VT* and *THI* were evaluated by a GLM procedure where *VT* and *THI* were set as the dependent variables. The climate conditions (*HW*, *NHW* and *TN*), parity as primiparous or multiparous, production level as low or high milk yield as described above, and cooling protocols as CC, EC90 and EC150 were set as the categorical independent variables.

The cow was included into the model and considered as random effect. The differences were analysed by the Tukey test, and the significances were set at a value of *p* < 0.01. The analysis was carried out using Stata software 11.2 (StataCorp 2011).

## Results

**Fig. 1:**
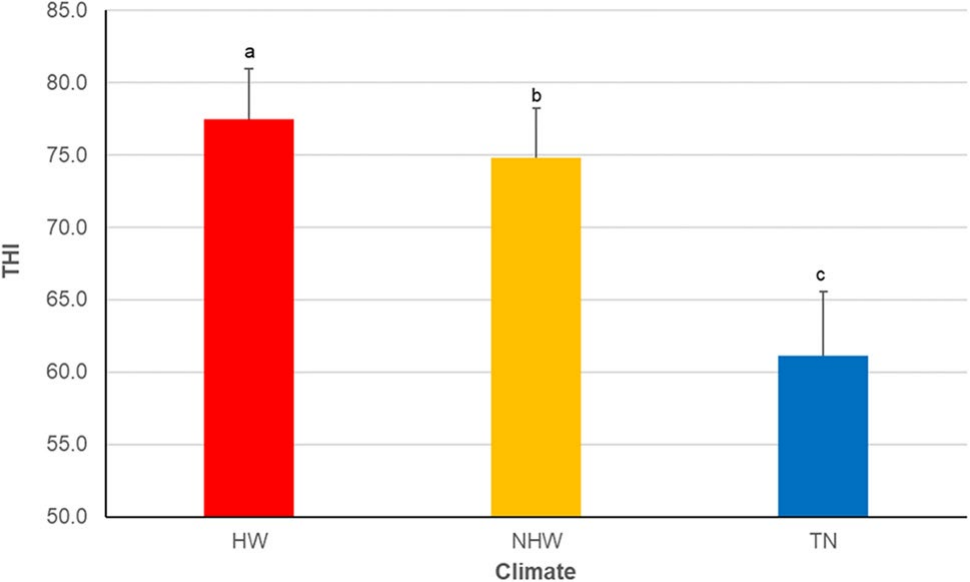
Mean values (± *SD*) of the temperature humidity index (*THI*) recorded in the pens during the trials: Heat wave days (*HW*); not heat wave days (*NHW*); thermo-neutral days (*TN*). Upper different letters indicate significant differences for *p* < 0.01

The *THI* recorded inside the barns ranged between 70.0 and 82.6, 66.2 and 80.0 and 52.4 and 69.9 during *HW*, *NHW* and *TN*, respectively. Daily mean values of *THI* during the three trials are shown in Fig. 1. The *THI* in *HW* (77.47 ± 3.48) was significantly higher than that recorded in *NHW* (74.79 ± 3.43) and *TN* (61.11 ± 4.42).

Table 2 shows the percentage of *THI* values falling into each of the four classes of *HL*. During *HW*, cows were exposed mainly to moderate and high *HL* conditions, for a lesser extent to low *HL* and never to null *HL* conditions. *NHW* cows were exposed for almost two-thirds of the time to moderate *HL*, for one third to low *HL*, for short time to null HL conditions and never to high *HL*. In the fall trial, cows spent almost all the time in the null *HL* class and only a little extent in low *HL* conditions.

**Table 2:** Distribution of *THI* values falling into each of the four classes of heat load (*HL*) during heat wave days (*HW*); not heat wave days (*NHW*) and thermo-neutral days (*TN*)

*THI* classes^a^	Climate		
	*HW*	*NHW*	*TN*
< 68 *THI*	0.0	4.2	90.6
68 < *THI* < 74	22.8	34.7	9.4
74 < *THI* < 80	43.7	61.1	0.0
*THI* > 80	33.4	0.0	0.0

^a^Classes of *THI* identify the following risk of *HL*: *THI* < 68 null; 68 < *THI* < 74 low; 74 < *THI* < 80 moderate; *THI* > 80 high

**Fig. 2:**
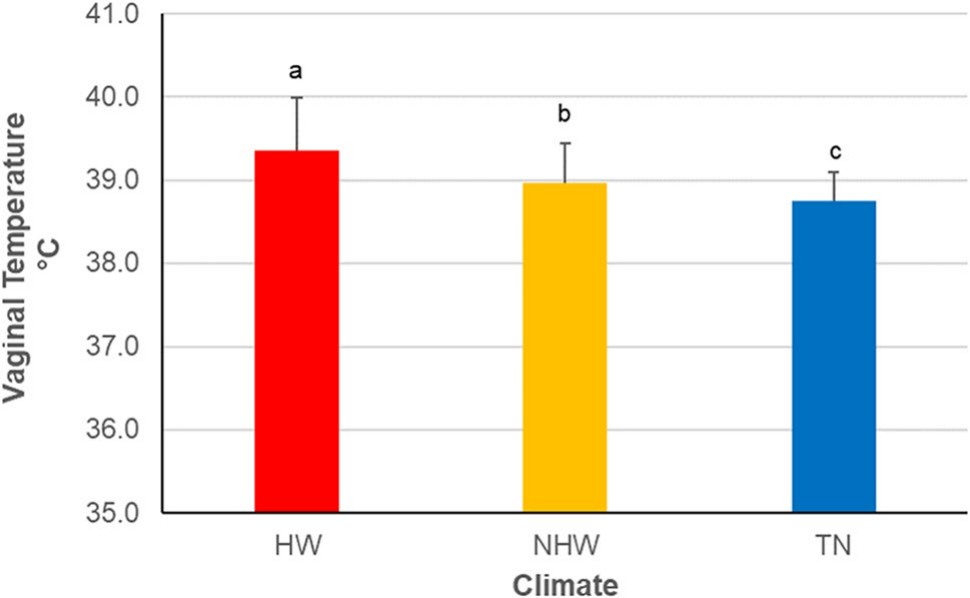
Mean values (± *SD*) of vaginal temperature (°C) of cows recorded during the trials: Heat wave days (*HW*); not heat wave days (*NHW*); thermo-neutral days (*TN*). Upper different letters indicate significant differences for *p* < 0.01

The *VT* of cows ranged between 37.6 and 41.4 °C, 37.5 and 40.6 °C and 37.1 and 40.7 °C in *HW*, *NHW* and *TN*, respectively. Mean values of *VT* in relation to climate conditions are shown in Fig. 2. The *VT* recorded during *HW* (39.36 ± 0.63) was above 39 °C that we assumed as the upper thresholds of normothermia, and it was higher (*p* < 0.01) than that observed in *NHW* (38.96 ± 0.48), which was below the upper threshold of normothermia. The lowest (*p* < 0.01) *VT* was observed in *TN* cows (38.75 ± 0.35).

Table 3 shows the *VT* in relation to parity and milk yield. During summer trials, primiparous cows showed lower *VT* compared to multiparous ones, and this feature was more pronounced in *HW* than in *NHW*. No difference was found between primiparous and multiparous cows in *TN*. Considering production level, high producing cows showed higher *VT* compared to low producing cows (*p* < 0.01) along all climate conditions, and the largest difference was observed in *TN*, whereas the lowest was recorded during HW.

**Table 3:** *Mean* values (± *SD*) of the vaginal temperature recorded in heat wave days (*HW*), not heat wave days (*NHW*), and thermo-neutral days (*TN*) in relation to production level and parity

	Vaginal temperature (°C)					
	Production level^a^		*p*-value	Parity^b^		*p*-value
	Low (*mean* ± *SD*)	High (*mean* ± *SD*)		1 (*mean* ± *SD*)	2 + (*mean* ± *SD*)	
*HW*	39.34 ± 0.62	39.38 ± 0.61	*	39.29 ± 0.62	39.41 ± 0.61	*
*NHW*	38.92 ± 0.47	39.00 ± 0.49	*	38.93 ± 0.46	38.98 ± 0.50	*
*TN*	38.64 ± 0.28	38.85 ± 0.37	*	38.73 ± 0.32	38.75 ± 0.36	NS

*NS* not significant

^*^ *p* < 0.01

^a^Production level of the cows: high (> 38/L/cow/day) and low (< 38/L/cow/day)

^b^Parity of cows: primiparous (1) and multiparous (2 +) cows

**Fig. 3:**
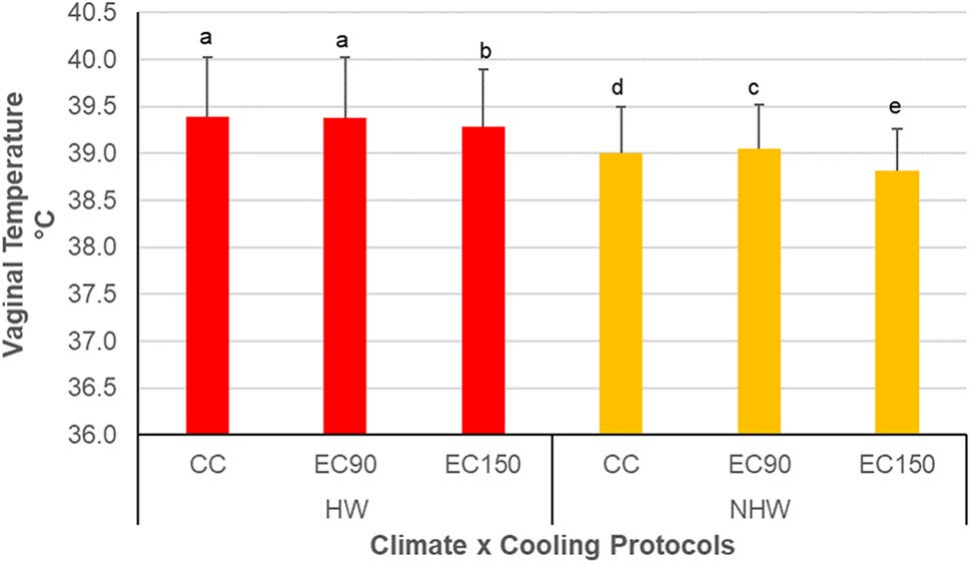
Mean values (± *SD*) of vaginal temperature (°C) of cows. Heat wave days (*HW*); not heat wave days (*NHW*); control cooling (CC) protocol based on five daily treatments; experimental cooling protocol EC90 based on CC plus two extra daily cooling treatments lasting in total 90′; experimental cooling protocol EC150 based on CC plus two extra daily cooling treatments lasting in total 150′. Upper different letters indicate significant differences for *p* < 0.01

The impact of cooling protocols on *VT* is reported in Fig. 3. Comparison of cooling protocols indicated a positive effect of the longer cooling treatments in that the EC150 reduced significantly *VT*, both in *HW* and *NHW*. On the other hand, compared to CC, the EC90 was not effective in reducing *VT* in *HW*. Surprisingly, during *NHW* days, the *VT* of EC90 was higher (*p* < 0.01) than that of CC cows. Finally, it has to be noticed that, despite the cooling treatments, during the *HW* the *VT* was always above the upper threshold of normothermia.

## Discussion

The *THI* values recorded inside the barns testified that trials were carried out at different degrees of hot conditions. In fact, during *NHW* cows were exposed from low to moderate *HL*, whereas in *HW*, they were exposed from moderate to high *HL* conditions. Weather alert may thus represent a proactive adaptation strategy informing farmers of the oncoming climatic risk, giving them the time to implement effective mitigation strategies. Forecasted alert services for heat stress in cattle are growing worldwide. The USDA’s Agricultural Research Service (ARS) has recently released a new smartphone application that forecasts 1 to 7 days’ conditions triggering heat stress in cattle, along with recommended actions that can mitigate negative impact on animals. Developing personalized proactive services at farm scale may be an adaptation strategy that may increase the resilience to heat waves, one of the most noxious climatic extreme events for livestock that will increase in frequency and intensity in temperate zones under the global warming scenario (Thornton et al. 2021).

Body temperature as well as respiration rate (panting frequency) provide valuable information on the relationship between an animal and its thermal environment (Polsky and Keyserlingk 2017). According to Vickers et al. (2010), the *VT* is highly correlated to rectal measures, and therefore it can be considered a good physiological parameter to establish body temperature. The physiological value of *VT* in Holstein lactating cattle ranges between 37 and 39 °C, depending primarily on *DIM*, season and time on the day (Kendall and Webster 2009). The average value (38.75 °C) of *VT* in our *TN* cows (*THI* = 61) was in line with the value of 38.78 ± 0·24 °C observed by Suthar et al. (2013) in lactating Holstein cows under similar climate conditions (*THI* = 62), and it was within the range of normothermia indicated above (37–39 °C). The *VT* (38.96 °C) recorded in *NHW* cows (*THI* = 75) was higher than that recorded in their *TN* counterparts, but it still remained within the range of normothermia indicated above (37–39 °C). Conversely, the *VT* (39.36 °C) recorded in *HW* cows (*THI* = 77) indicated a positive thermal balance, in that cows monitored during heat wave loaded more heat of that they were able to dissipate. Therefore, regardless the cooling protocols applied, the hot conditions in *HW* triggered a certain level hyperthermia.

Several intrinsic factors contribute to determine the susceptibility of dairy cattle to HL (West 2003), and among them, our study focused on parity and production level. Primiparous cows showed lower *VT* both under moderate hot conditions in *NHW* and severe hot conditions in *HW*. This feature would indicate a greater ability of primiparous cows to dissipate and/or not to accumulate heat compared to multiparous ones when exposed to hot environment. Conversely, parity of cows was not associated to different *VT* during the *TN* trial. These outputs confirm previous data indicating a greater thermo-tolerance of primiparous cows (Bernabucci et al. 2014). Becker et al. (2020) suggested that this aptitude might be related to lower milk yield of younger cows.

According to West (2003), the greater capacity of primiparous cows to avoid hyperthermia might instead be due to the favourable ratio between the surface and mass of the body. However, Castro-Montoya and Corea (2021) found that rectal temperature of primiparous Holstein × Brahman in hot environment was higher than that of a multiparous counterpart even if primiparous yielded less milk than multiparous. These authors explained their result with the negligible difference in body weight between their primiparous and multiparous cows and concluded that, in the condition of their study, the recognized greater capacity of primiparous cows to dissipate heat due to their greater surface area to body mass ratio was minimised.

A Chinese study reported that multiparous cows are more sensitive to *HL* compared to younger cows. In their trial, multiparous cows (third parity and higher) exhibited a lower *THI* threshold for rectal temperature than cows in the first or second lactation (Yan et al. 2021).

Milk yield of primiparous and multiparous cows enrolled in our study was similar among the trials in *NHW* (36.9 ± 4.5 vs 39.5 ± 5.6; *p* = 0.727), *HW* (37.6 ± 4.9 vs 38.6 ± 5.4; *p* = 0.993) and *TN* (37.4 ± 3.5 vs 38.7 ± 4.9; *p* = 0.990). We did not weight primiparous and multiparous cows enrolled in the study, but assuming that body weight of our primiparous cows was about 15% lower than that of multiparous as reported for Holstein dairy cows (Neave et al. 2017) and, in the light of comparable production levels, we may speculate that, in the conditions of our study, the lower *VT* of primiparous cows might have depended on the greater capacity of younger animals in dissipating heat as consequence of the favourable ratio between the surface and mass of the body.

High yielding cows are indicated to be more susceptible to *HL* if compared to low-producing ones. West (2003) reported that this would be due to their need to eat more feed that would thus generate a greater production of metabolic heat. In our study, we observed higher *VT* in high-yielding cows along all climate conditions tested. However, the difference between high- and low-yielding cows was greatest in *TN* and lowest in *HW*. These data would suggest that the metabolic heat generated by milk synthesis would represent the major factor to explain the higher body temperature of high-yielding cows in *TN*, whereas, during high hot conditions in *HW*, the heat surrounding the animal would attenuate the role of the metabolic heat generated for milk synthesis in affecting thermal balance. Further studies are needed to clarify the contribution of metabolic heat related to milk synthesis and of climate conditions in the thermal balance of dairy cattle.

Finally, previous studies demonstrated that the regulation of body temperature recognizes also a genetic component that can be heritable (Dikmen et al. 2012) and that therefore cows may be selected for heat tolerance with evidence that animal selected for this trait may have lower core body temperatures when exposed to heat condition (Garner et al. 2016). Recently, Finocchiaro et al. (2022) carried out a genetic evaluation of heat stress tolerance on Italian Holstein cattle population reporting a heritability for this trait of 0.16 and a negative correlation with milk yield (− 0.45), indicating that heat tolerance should not be used as the sole criterion for selection. Furthermore, since April 2022 the Italian Holstein, Brown and Jersey breeder association (ANAFIBJ) releases the Index Heat Tolerance (*IHT*) for all bulls authorized for artificial insemination in Italy, with daughters in Italy or with a genotype available, and for all genotyped females. The index is expressed with a mean of 100 and a standard deviation of 5, and animals with an index greater than 100 would be animals whose offspring has to be considered genetically superior for heat tolerance. Unfortunately, the *IHT* values were not available at the time of our trial (2019) and thus we were not able to consider it as an additional criterion to balance the experimental groups or as a factor to include in our statistical model. However, in the light of what has been reported above, consideration of the genetic relationship among cows and the availability of genetic indexes categorizing cows for thermotolerance will surely accelerate the scientific progress in this field and the implementation of adaptation strategies to the ongoing increase of ambient temperatures.

All the cooling protocols tested in the study were effective in guaranteeing normothermia (< 39 °C) under moderate *HL* conditions in *NHW* (daily average *THI* = 74.8), whereas none of them prevented hyperthermia during *HW* (daily average *THI* = 77.5). Compared to the control cooling protocol, 90′/day of additional cooling treatments (EC90) did not reduce *VT* and, in contrast on what was expected, values of *VT* in EC90 cows were surprisingly higher in *NHW*. The EC150 was more effective in mitigating *HL* compared to other protocols. However, the advantage of EC150 was small during *HW*, and it was not sufficient at avoiding completely the risk of hyperthermia (*VT* was still above 39 °C).

A Mexican study assessed the effectiveness of different cooling protocols in a commercial farm of Holstein cattle (Avendaño-Reyes et al. 2010). A standard cooling protocol based on treatments of half hour applied before morning (at 7:00 AM) and afternoon (at 5:00 PM), milking was compared with three experimental protocols based on the standard protocol to which extra treatments of 1 h each were added, in the morning (at 11:00 AM), in the evening (at 11:00 PM) and both in morning and evening (both at 11:00 AM and at 11:00 PM). The authors observed a positive effect on rectal temperature and respiration rate of the two extra treatments, whereas a single additional treatment did not highlight a clear effect.

Pinto et al. (2019) compared respiration rate of cows cooled three times in the waiting parlour (control protocol) with that of cows cooled with the control protocol to which five treatments were added during the rest of the day. The authors observed that cows cooled 8 times/day showed lower respiration rate and lower heat load.

Finally, Tresoldi and colleagues (2018) studied strategies aimed at improving cow cooling and water-use efficiency. The application of the same water volume more often reduced respiration rate by 7 breaths/min on restrained cattle over a 45-min period. In contrast, spraying cows for longer time, increasing time on or reducing the time off (using more water) reduced both respiration rate by 7 breaths/min and body temperature by at least 0.1 °C.

Our results would suggest that two additional treatments to a daily cooling protocol based on five treatments could not be sufficient to mitigate *HL* if an adequate duration of treatment is not considered. Two 45-min extra cooling treatments did not reduce vaginal temperature with respect to control protocol and therefore, in this case, the water and energy needed for extra cooling operations would not be justified. Conversely, the positive effects obtained by longer treatments (two 75-min extra cooling) would justify, at least partially, the extra water and energy use.

In conclusion, forecasted weather bulletins for heat stress can alert farmers of the incoming risk and give them the time to plan actions to mitigate the negative effects of heat load, especially in anticipation of heat waves. Providing farmers with information on *HL*-related risk along with suggestions on the best options for cooling management may represent a strong proactive behaviour for adaptation to climate change that will help them to face heat load challenges and, at the same time, improve environmental sustainability of the milk production chain. Evaporative cooling systems reduce the impacts of *HL* in dairy cattle, ensuring animal welfare and reducing milk loss. However, during climatic extremes such as heat waves, the effectiveness of cooling systems is reduced even if intensive, and dairy cattle are still at risk of hyperthermia. The development of operative cooling protocols based on *HL*-related risk and on the characteristics of the animals may further improve their effectiveness, reduce economic losses and increase the efficiency in the use of water and energy.

## Data Availability

No data were deposited in an official repository. The study findings are available on request.

## Declarations

### Disclaimer

This manuscript reflects only the authors’ views and opinions; neither the European Union nor the European Commission can be considered responsible for them.
